# Psychometric Evaluation of the Revised Suicide Crisis Inventory (SCI‐2) in Germany: Factor Structure, Reliability, and Validity in an Online and Outpatient Sample

**DOI:** 10.1002/cpp.70106

**Published:** 2025-06-24

**Authors:** Laura Melzer, Carola Claus, Nelia Posen, Thomas Forkmann, Megan L. Rogers, Tobias Teismann

**Affiliations:** ^1^ Mental Health Research and Treatment Center Ruhr‐Universität Bochum Bochum Germany; ^2^ Department of Clinical Psychological Intervention University of Wuppertal Wuppertal Germany; ^3^ Department of Clinical Psychology and Psychotherapy University of Duisburg‐Essen Essen Germany; ^4^ Department of Psychology Texas State University San Marcos Texas USA; ^5^ DZPG (German Center for Mental Health), Partner Site Bochum/Marburg Augsburg Germany

**Keywords:** psychometric evaluation, suicide crisis syndrome, suicide risk assessment

## Abstract

**Background:**

The suicide crisis syndrome (SCS) is considered a cognitive and affective state preceding a suicide attempt. Previous studies have demonstrated its predictive validity and have shown it to be a uniform disorder entity in various countries worldwide; however, research in Europe remains limited. The aim of this study was therefore to contribute to a cross‐cultural perspective on the SCS and to examine its factor structure, reliability, and validity in Germany.

**Methods:**

Data of *N* = 1157 participants were collected between October 2022 and December 2024 cross‐sectionally from a German adult outpatient sample (*n* = 586; 65% female; age: *M*[SD] = 36.31[12.80], range: 18–68) and a German adult online sample (*n* = 571; 74% female; age: *M*[SD] = 24.69[7.20], range: 18–67). Participants completed the German version of the revised 61‐item Suicide Crisis Inventory (SCI‐2‐G) and other instruments (e.g., SIBS, BDI‐II, and DASS) to measure convergent and concurrent criterion validity. Statistical analyses included confirmatory factor analyses (CFAs) to confirm the proposed factors (entrapment, affective disturbance, loss of cognitive control, hyperarousal, and social withdrawal).

**Results:**

The SCI‐2‐G total score demonstrated excellent internal consistency, good convergent, and moderate concurrent criterion validity in relation to stress, depression, anxiety, suicidal ideation, and lifetime suicide attempts. The CFA showed good model fit for the online sample and adequate‐to‐good fit for the outpatient sample, with the five‐factor model consistently outperforming the one‐factor model.

**Conclusion:**

CFA yielded adequate‐to‐good model fit depending on the sample. Analyses indicate that the SCI‐2 is a valid measurement tool in Germany. The five‐factor solution is suggested to have higher clinical utility than a one‐factor solution, as it reflects the current formulation of the proposed SCS diagnosis. Future studies should expand the cross‐cultural perspective with longitudinal studies across diverse clinical and nonclinical samples.

**Summary:**

- First validation of the German revised version Suicide Crisis Inventory (SCI‐2‐G).
- Confirmatory factor analyses support the five‐factor solution of the scale.
- Good scale properties for an online and outpatient sample in Germany.

## 1 Introduction

Suicide remains one of the leading causes of death worldwide (WHO 2024), accounting for 1% of all deaths in Germany (DeStatis 2024). Despite fluctuations in suicide rates over recent decades, it continues to be a major public health concern with over 720,000 deaths by suicide each year (WHO 2024). In 2023, the German Federal Statistical Office reported 10,300 suicide deaths in Germany, indicating an increase of 1.8% compared to the previous year, with a notable rise (+8.0%) among women (DeStatis 2024).

Early identification of risk and protective factors may thus be crucial for implementing effective preventative and safety measures to address this pressing issue (e.g., Ryan and Oquendo 2020). Suicide risk assessment primarily focuses on traditional risk factors such as a history of suicide attempts (Bostwick et al. 2016), mental illness (Nock et al. 2008), or suicidal ideation (Hubers et al. 2018). However, the predictive accuracy of these traditional risk factors is only slightly better than chance (Franklin et al. 2017). Furthermore, common approaches to risk assessment rely heavily on the disclosure of suicidal ideation to clinicians, despite meta‐analytic nondisclosure rates of 50%–60%, often due to fear of potential consequences (Hallford et al. 2023).

Recognizing these limitations, the critical role of warning signs for acute suicidality has been recently highlighted (Tucker et al. 2015; Galynker 2017), as initially introduced by an expert consensus (Rudd et al. 2006). In contrast to long‐term risk factors associated with suicidal thoughts and behavior (e.g., a mental health diagnosis), warning signs reflect the current acute state of the suicidal person (Rudd et al. 2006). Drawing an analogy to a heart attack, which “is predicted by warning signs that reflect lack of myocardial perfusion or ischemia” (e.g., crushing chest pain), Galynker (2017, 3) proposed the suicide crisis syndrome (SCS) as a suicide‐specific syndrome indicative of an acute suicidal crisis. Within the so‐called narrative crisis model (NCM) of suicide (Bloch‐Elkouby et al. 2022, 2024; Rogers et al. 2024), the SCS represents the culmination of internal and interpersonal processes through which chronic, long‐term risk factors—such as hopelessness, impulsivity, or perfectionism—become acutely destabilizing. The NCM thus provides a framework for understanding how enduring vulnerabilities may translate into imminent suicidal risk. Unlike in traditional models, the SCS does not depend on suicidal ideation but instead captures a distinct negative mental and affective state. It has been shown to be predictive of suicidal behavior at 1 month follow‐up (Galynker et al. 2017; Bloch‐Elkouby et al. 2021; Melzer et al. 2024; Rogers, Vespa, et al. 2021).

After a period of 15 years of iterative development, the SCS formulation was finalized and consists of the following constructs (Galynker et al. 2024): (A) Entrapment describes an intense feeling of being “trapped in a situation experienced both as intolerable and inescapable”; (B1) affective disturbance displays as emotional pain, rapid spikes of negative emotions, extreme anxiety, and acute anhedonia; (B2) loss of cognitive control includes rumination, cognitive rigidity, ruminative flooding, and failed thoughts suppression; (B3) hyperarousal is expressed by agitation, hypervigilance, irritability, and insomnia; and (B4) social withdrawal results from the avoidance of social contact and feelings of isolation. For a diagnosis, Criterion A and at least one symptom of each Criteria B1–B4 must be met (Bafna et al. 2022).

To measure the SCS, a self‐report instrument, called the Suicide Crisis Inventory (SCI), was originally developed with 49 items (Barzilay et al. 2020). In accordance with the latest SCS formulation, the SCI was revised and now contains 61 items across five subscales (SCI‐2; Bloch‐Elkouby et al. 2021). A growing body of literature has investigated the psychometric properties of the SCI‐2 and provided support for both its unidimensional disorder entity and five‐factor structure (Bloch‐Elkouby et al. 2020; Bloch‐Elkouby et al. 2021; Chistopolskaya et al. 2022; Menon et al. 2022, 2024; Park et al. 2023; Wu et al. 2022), with consistent superiority of the five‐factor solution. Although these mixed findings seem contradictory, the authors argue that the SCS criteria “incorporate five empirically validated domains, which together constitute a unidimensional syndrome” (Galynker et al. 2024).

The internal consistency of the SCI‐2 total score was excellent (*α* = 0.97–0.98), and subscale scores ranged from *α* = 0.82–0.96 (Bloch‐Elkouby et al. 2021; Menon et al. 2022; Wu et al. 2022). Previous studies have also shown meaningful associations with psychological distress, including depression (e.g., Wu et al. 2022), anxiety (Park et al. 2023), suicidal ideation (concurrent, past month, and lifetime) (e.g., Rogers, McMullen, et al. 2023), and suicide attempts (past month and lifetime) (e.g., Bloch‐Elkouby et al. 2021; Cohen et al. 2022; Menon et al. 2022; Wu et al. 2022).

There has been ongoing debate about the pros and cons of implementing a suicide‐specific diagnosis (Berman and Silverman 2023; Cohen et al. 2023; Joiner et al. 2018; Oquendo and Baca‐Garcia 2014; Rogers, Chu, and Joiner 2019; Wortzel et al. 2018). Such syndromes may help identify individuals in an imminent suicidal state, but the “illusion of short‐term prediction” (Simon 2006, 296) remains a critical limitation. A syndrome similar to the SCS is the Acute Suicidal Affective Disturbance (ASAD) introduced by Joiner et al. (Tucker et al. 2016; Rogers et al. 2017; Stanley et al. 2016). Differences in symptom dimensions and predictive validity were demonstrated by Rogers, Jeon, et al. (2023), who compared ASAD to SCS. Although a validation study of the German version of the ASAD Inventory‐Lifetime (ASADI‐L; Rogers and Joiner 2018a; Rogers, Hom, and Joiner 2019), a German instrument to assess ASAD, was recently published (Claus et al. 2025), no article exists on the validation of the German version of the revised SCI (SCI‐2‐G), as only the psychometric properties of its previous version (SCI‐G; Otte et al. 2020) have been evaluated in a forensic German sample thus far.

Despite promising psychometric evidence from various countries worldwide, further international validation—especially in Europe—is needed for the revised instrument to assess the SCS (Melzer et al. 2024). The growing cross‐cultural evidence on the SCI‐2 supports the conceptualization of the SCS as a unified syndrome comprising five symptomatic dimensions in the United States (e.g., Bloch‐Elkouby et al. 2021), India (Menon et al. 2022, 2024), Taiwan (Wu et al. 2022), Brazil (Peper‐Nascimento et al. 2024), Korea (Park et al. 2023), and Russia (Chistopolskaya et al. 2022). The cross‐national presence of the SCS was further demonstrated in a large community‐based study during the COVID‐19 pandemic across 10 countries (Rogers, McMullen, et al. 2023). Germany (4.5%) ranked at the lower end of the proportion of participants who met the SCS criteria, with rates ranging from 3.6% in Israel to 16.2% in Poland. The severity of SCS symptoms was significantly lower in Germany (*n* = 532, *M* = 64.93, SD = 46.23) (and India) compared to all other states, except for Israel. Additionally, sociodemographic data in Germany and elsewhere revealed lower SCS severity in older, married, and cisgender men compared to younger, single, and cisgender women. These findings emphasize the importance of considering both cultural and demographic factors when examining the SCS.

To our knowledge, this is the first study to investigate the validity and psychometric properties of the revised version of the SCI (SCI‐2) in a German online and outpatient sample. The first aim was to translate the questionnaire into German following standard procedures. The second aim was to conduct a psychometric validation of this German version of the instrument. Based on the previous literature, we hypothesized that (1) the CFA will indicate strong model fit for one‐ and five‐factor solutions for both the online and outpatient samples, (2) the reliability of the instrument will be good to excellent, and (3) the SCI‐2‐G will have good convergent and concurrent criterion validity in relation to depression, anxiety, stress, suicidal ideation, and suicidal behavior.

## 2 Methods

### 2.1 Participants

Data were obtained from two samples in Germany between October 2022 and December 2024. Descriptive statistics for sociodemographic characteristics and suicidality are presented in Table 1.

**TABLE 1:** Demographics and suicidality.

	*n* (%)	Online Sample (*n* = 571)	Outpatients (*n* = 586)	Group difference
Age		*M*[SD] = 24.7[7.2]	*M*[SD] = 36.3[12.8]	*t*(1155) = −18.96***
Gender	Male	103 (18.0%)	206 (35.2%)	*χ* ^2^(2) = 82.35***
	Female	422 (73.9%)	380 (64.8%)	
	Diverse	46 (8.1%)	*NA*	
SI recent week		227 (39.8%)	228 (39.0%)	*t*(1155) = 3.92***
SA recent month		13 (2.3%)	2 (0.3%)	
SA lifetime		81 (14.2%)	46 (7.8%)	*χ* ^2^(1) = 11.88***
SCS	Cutoff ≥ 164	86 (15.1%)	109 (18.6%)	*t*(1155) = −8.79***

Abbreviations: SA = suicide attempts, SCS = suicide crisis syndrome, SI = suicidal ideation.

*** Group difference between the online and outpatient sample is significant at level *p* < 0.001.

#### 2.1.1 Sample 1 (Online Sample)

The online sample comprised *n* = 855 participants. Of those, *n* = 571 individuals were included in the final sample. Participants were excluded if they were under 18 years old (*n* = 2) or exhibited specific response tendencies, such as not completing the full survey battery (*n* = 226), speeding (*n* = 50) measured using the Relative Speed Index (RSI; Leiner 2019), or long‐string (*n* = 6), that is, the repeated selection of identical response options (Curran 2016). Data collection was conducted between October 2022 and May 2023. Recruitment took place via the study participant platform of the local university and via various social networks. The study flyer explicitly mentioned the investigation of protective and risk factors for suicidal experiences. All participants were informed about the study procedures and gave their informed consent. Psychology students were each compensated with one course credit. In the event of an acute psychological crisis, participation was not recommended, and contact addresses for support services were provided. Participants answered self‐report online questionnaires on the Qualtrics web platform (Qualtrics 2024).

Almost three quarters of the participants in the final sample identified as female (73.9%; *n* = 422), 18.0% as male (*n* = 103), and 8.1% (*n* = 46) as diverse. The mean age was 24.69 years (SD = 7.20) ranging from 18 to 67 years. More than half of the participants, *n* = 360 (63.1%), had never been diagnosed with a mental health diagnosis before, *n* = 140 (24.5%) were currently diagnosed with a mental disorder, and *n* = 71 (12.4%) had been diagnosed in the past. Accordingly, more than half of the sample had not previously received psychotherapy (*n* = 313, 54.8%). Current psychotherapy was reported by *n* = 107 (18.7%) and past psychotherapy by *n* = 151 (26.5%). Concurrent suicidal ideation, as measured by the Suicide Ideation and Behavior Scale (SIBS; Teismann et al. 2021), was reported by *n* = 227 (39.8%, with SIBS score ≥ 1). Eighty‐one participants (14.2%) disclosed a history of suicide attempts, 13 of them (2.3%) in the last 4 weeks. The ethics committee of the Faculty of Psychology at Ruhr University Bochum approved the study procedures and data collection (797).

#### 2.1.2 Sample 2 (Outpatient Sample)

Sample 2 comprised a total of *n* = 586 (65% female; age: *M*[SD] = 36.31[12.80], range: 18–68) outpatients who underwent cognitive‐behavioral psychotherapy at a German university outpatient clinic between April 2023 and December 2024. Before starting therapy, all patients took part in a computer‐based diagnostic assessment with self‐report questionnaires. The most common main diagnoses according to the International Classification of Diseases (ICD; WHO 1992) were neurotic, stress‐related, and somatoform disorders (ICD‐10, F4: 53%), affective disorders (ICD‐10, F3: 34%), or personality disorders (ICD‐10, F6: 6%). Two hundred twenty‐eight patients (39% with a SIBS score ≥ 1; Teismann et al. 2021) reported suicidal ideation prior to treatment. Additionally, 46 patients reported a history of suicide attempts, with two occurring within the past month. All patients gave informed consent and completed the questionnaires, with no missing data. The local ethics committee approved the study procedures and data collection (318/2016).

The samples differed significantly in terms of age, *t*(1155) = −18.96, *p* < 0.001, and suicidal ideation, *t*(1155) = 3.91, *p* < 0.001, with the outpatient sample being on average older (*M*_*age*_ = 36.31; SD_*age*_ = 12.80) and reporting higher suicidal ideation (*M*_*SI*_ = 2.27, SD_*SI*_ = 4.54) than the online sample (*M*_*age*_ = 24.69, SD_*age*_ = 7.20; *M*_*SI*_ = 1.39, SD_*SI*_ = 2.92). Cohen's *d* indicated a large negative effect for age (*d* = −1.12) and a small effect for suicidal ideation (*d* = 0.23). Further, there were significant group differences between the samples in terms of gender (*χ* ^2^[2] = 82.35, *p* < 0.001, Cramer's *V* = 0.267 [small]), lifetime suicide attempts (*χ* ^2^[1] = 11.88, *p* < 0.001, Cramer's *V* = 0.101 [small]), and current mental health diagnosis (*χ* ^2^[1] = 704.91, *p* < 0.001, Cramer's *V* = 0.781 [large]). Group differences for past‐month suicide attempts were not calculated due to the low prevalence.

### 2.2 Measures

#### 2.2.1 The Revised SCI (SCI‐2‐G) in German

The translated German version of the revised SCI (SCI‐2; Bloch‐Elkouby et al. 2021) was utilized to assess the severity of the SCS on all five dimensions: entrapment (10 items), affective disturbance (18 items), loss of cognitive control (15 items), hyperarousal (13 items), and social withdrawal (5 items). The SCI‐2 consists of 61 items on a 5‐point Likert scale ranging from 0 (*not at all*) to 4 (*extremely*). The questions refer to an emotional low point (“feeling your worst”) during the last few days. The original version of the SCI‐2 demonstrated excellent internal consistency (Cronbach's *α* = 0.97; Bloch‐Elkouby et al. 2021).

The English version of the SCI‐2 was translated into German following standard procedures. Initially, all items from the previous German version (SCI‐G; Otte et al. 2020) were incorporated, and additional SCI‐2 items selected from instruments (for overview, see Bloch‐Elkouby et al. 2021) available in existing German versions were adopted. Items newly created by the authors of the original SCI‐2 (Bloch‐Elkouby et al. 2021) were translated into German by a doctoral student in clinical psychology also undergoing psychotherapist training with C1‐level English proficiency. The back‐translation was carried out by a co‐author of this study, an expert in suicide prevention and professor of clinical psychology. A native speaker then reviewed the back‐translations for accuracy and clarity. Finally, the translations were compared with a version utilized by another German research team evaluating the short form of the SCI‐2, the so‐called SCI‐2‐SF (Spangenberg et al. 2025). At the same time, another German research group completed an independent translation without being aware of each other's efforts. After this study was conducted, the translation was compared with the version used in the large cross‐sectional study (Rogers, McMullen, et al. 2023). The independent translations are largely comparable, with minor differences in wording for some items that do not affect meaning.

The following measures were used to assess validity in one or both samples:

#### 2.2.2 Suicidal Ideation and Behavior

The SIBS (Teismann et al. 2021) measures current and lifetime suicidal experiences with nine items, of which six items refer to suicidal ideation, intentions, and impulses on a 6‐point Likert scale (0—*never* to 5—*many times every day*). All items begin with the statement “During the last four weeks …” and include self‐reports such as “I thought it would be better if I wasn't alive.” Higher sum scores, ranging from 0 to 30, indicate a higher severity of suicidal thoughts within the last month. Three further items comprise a dichotomous (yes/no) assessment of previous and lifetime suicide attempts and number of suicide attempts. The reliable and valid German self‐report instrument demonstrated excellent internal consistency in both its psychometric evaluation (*α* = 0.92; Teismann et al. 2021) and the online sample (*α* = 0.91) and good internal consistency in the outpatient sample (Cronbach's *α* = 0.82).

#### 2.2.3 Depression

In the online sample, the depression module of the Patient Health Questionnaire (PHQ‐9, Kroenke et al. 2001) was used to measure various symptoms of depression with nine items on a 4‐point Likert scale ranging from 0 (*not at all*) to 3 (*almost every day*) within the last two weeks. Higher values of the sum score correspond to a higher level of depression. The internal consistency of the total scale was excellent in the online sample (Cronbach's *α* = 0.90), in line with previous studies (*α* = 0.86–0.89; Kroenke et al. 2001).

In the outpatient sample, the German revised version of the Beck Depression Inventory (BDI‐II; Hautzinger et al. 2006) was used to measure the severity of depression with 21 items on a 4‐point Likert scale ranging from 0 to 3 (individual item answers) within the past 2 weeks. Suicidal thoughts or wishes are assessed with Item 9. The internal consistency of the total scale was excellent in the outpatient sample (Cronbach's *α* = 0.91), exceeding reported values in diverse samples of *α* ≥ 0.84 (Kühner et al. 2007).

#### 2.2.4 Anxiety and Stress

The Depression Anxiety Stress Scale (DASS‐42‐G, German translation by Nilges and Essau 2015; Lovibond and Lovibond 1995) captures these respective emotional states with 42 items on a 4‐point Likert scale from 0 (*did not apply to me at all*) to 3 (*applied to me very much or most of the time*). It consists of three subscales, each containing 14 items. Higher sum scores indicate higher severity. The depression scale (DASS‐D) measures low mood and negative affect, the anxiety scale (DASS‐A) assesses physiological and subjective effects of anxiety, and the stress scale (DASS‐S) measures stress sensitivity. The internal consistency for each scale was good to excellent in the outpatient sample (depression: *α* = 0.95; anxiety: *α* = 0.89; stress: *α* = 0.93), with stronger reliability than in a previous study (*α* = 0.76–0.88; Nilges and Essau 2015).

### 2.3 Statistical Analysis

Sociodemographic characteristics of both samples were calculated using descriptive statistics. Group differences between the online and outpatient samples were assessed using an independent samples *t* test for age, and crosstabulations with chi‐square tests for gender and mental health diagnoses. Effect sizes were calculated using Cohen's *d* for *t* tests, with *d* ≥ 0.80 indicating a large effect, and Cramer's *V* for chi‐square tests, with values above 0.50 suggesting a large effect (Cohen 1988). These analyses were conducted using SPSS 29. Reverse‐coded items of the SCI‐2‐G were recoded for all analyses. Data were further analyzed using different packages in R (Version 4.4.1) (R Core Team 2019), specifically the *lavaan* (Rosseel 2012) package for CFA analyses and *psych* (Revelle 2024) for internal consistency analyses. Scale reliability was assessed using Cronbach's *α*, with values ≥ 0.70 indicating good internal consistency (Nunnally 1975). To determine the suitability of the SCS data for confirmatory factor analysis (CFA), the Kaiser–Meyer–Olkin (KMO) measure of sampling adequacy (Kaiser and Rice 1974) and Bartlett's test of sphericity (Bartlett 1951) were used. The previously proposed one‐ and five‐factor structures of the original SCI‐2 were then re‐evaluated for the German samples conducting CFAs. The five‐factor solution contained the respective subscales (*entrapment*, *affective disturbance*, *loss of cognitive control*, *hyperarousal*, and *social withdrawal*), whereas the one‐factor solution pertained to all items loading onto a single factor. Diagonally weighted least squares (DWLS) estimation, as recommended for Likert scales and nonnormal distributions (Mooney and Duval 1993; Mîndrilă 2010), was used for the CFA to ensure comparability with previous studies (e.g., Bloch‐Elkouby et al. 2021; Wu et al. 2022). Model fit was assessed following established guidelines (Hu and Bentler 1999), utilizing the chi‐square statistic (*χ* ^2^), comparative fit index (CFI), Tucker–Lewis index (TLI), root mean square error of approximation (RMSEA), and standardized root mean squared residual (SRMR). A good model fit was characterized by a nonsignificant *χ* ^2^ statistic, CFI, and TLI values of 0.95 or higher, RMSEA of 0.06 or lower, and SRMR of 0.08 or lower. Due to its high sensitivity to sample size, the *χ* ^2^ statistic test should be interpreted with caution (Browne and Cudeck 1993). The robust chi‐square difference test was used to compare model fit of the one‐factor and five‐factor models. Bivariate correlations between the German SCI‐2 total and subscale scores with other relevant instruments were calculated to assess convergent validity using Spearman's rho due to the nonnormality and/or nonlinearity of the data distribution. Concurrent criterion validity was assessed by examining the correlation between the SCI‐2‐G total score and suicidal ideation, as well as point‐biserial correlations with past‐month and lifetime suicide attempts. The outpatient sample contained no missing data, as only complete cases were included initially. After excluding individuals who did not finish the survey battery, there were no missing data in the online sample.

## 3 Results

Total scores of the SCI‐2‐G ranged from 14 to 225 (*M* = 120.05, SD = 45.38) in the outpatient sample and from 6 to 227 (*M* = 94.59, SD = 56.70) in the online sample. The group difference was significant, *t*(1155) = −8.79, *p* < 0.001. One hundred nine outpatients (18.6%) and 86 online participants (15.1%) scored at or above an established cutoff score of 164, which is deemed to be indicative of the presence of the SCS (see Bloch‐Elkouby et al. 2021).

### 3.1 Factor Structure

Both samples demonstrated significant correlations between variables, confirming their suitability for factor analysis by an excellent KMO statistic (online: 0.98; outpatient: 0.96) and significant Bartlett's test of sphericity (online: *χ* ^2^[1830] = 26,902.94, *p* < 0.001; outpatient: *χ* ^2^[1830] = 19,886.62, *p* < 0.001). CFA results are presented in Table 2.

**TABLE 2:** Fit indices for models tested with CFA for each sample (online sample: *n =* 571; outpatient sample: *n* = 586).

Fit indices	One‐factor CFA		Five‐factor CFA		
	Online sample	Outpatient sample	Online sample	Outpatient sample	
*χ* ^2^	5216.42***	8614.20***	3985.37***	6442.71***	*χ* ^2^ nonsignificant is a close fit
CFI	**0.995**	**0.974**	**0.997**	**0.982**	CFI ≥ 0.95 indicates a good fit
TLI	**0.995**	**0.973**	**0.997**	**0.981**	TLI ≥ 0.95 indicates a good fit
RMSEA	**0.058**	0.081	**0.047**	0.067	RMSEA ≤ 0.06 indicates a good fit
SRMR	**0.053**	**0.075**	**0.048**	**0.067**	SRMR ≤ 0.08 indicates a good fit

*Note:* All factor loadings *p* > 0.001. Cutoff values for fit indices are suggested by Hu and Bentler (1999). Good fit is shown in bold print. *χ* ^2^ is sensitive to sample size; with large *N*, significant values may not indicate misfit and should be interpreted with other fit indices.

Abbreviations: *χ* ^2^ = chi‐square; CFA = confirmatory factor analysis; CFI = comparative fit index; RMSEA = root mean square error of approximation; SRMR = standardized root mean squared residual; TLI = Tucker–Lewis index.

*** Chi‐square test is significant at level *p* < 0.001.

#### 3.1.1 Online Sample

A one‐factor solution fit the data well based on most fit indices (CFI = 0.995, TLI = 0.995, RMSEA = 0.058, SRMR = 0.053). However, the chi‐square test was significant due to the large sample size, *χ* ^2^(1769) = 5216.42, *p* < 0.001. The five‐factor solution also demonstrated good model fit (CFI = 0.997, TLI = 0.997, RMSEA = 0.047, SRMR = 0.048), except for the significant chi‐square test, *χ* ^2^(1759) = 3985.37, *p* < 0.001. The CFA calculated with the DWLS estimator supported both the one‐factor and five‐factor solutions, with the five‐factor model showing superior fit compared to the one‐factor model (Δ*χ* ^2^[10] = 1231.05, *p* < 0.001). In both models, all items loaded > 0.40 on their respective factors, except for the reverse‐coded Items 24r, 34r, 37r, and 9 from the cognitive rigidity scale of the *loss of cognitive control* dimension. After removing these items, no major improvement in global model fit was observed. In the five‐factor model, all latent factors were significantly related to each other (*p* < 0.001). Standardized factor loadings and covariances are shown in Tables 3 and 4.

**TABLE 3:** Standardized factor loadings of all items.

Factor/item	One‐factor model		Five‐factor model	
	Online sample	Outpatient sample	Online sample	Outpatient sample
Entrapment (10 items)				
Item 2	0.85	0.73	0.87	0.76
Item 4	0.74	0.65	0.76	0.69
Item 15	0.87	0.74	0.90	0.78
Item 19	0.80	0.70	0.82	0.74
Item 25	0.78	0.66	0.80	0.70
Item 27	0.90	0.81	0.92	0.86
Item 35	0.90	0.81	0.92	0.85
Item 39	0.91	0.80	0.93	0.84
Item 56	0.91	0.85	0.93	0.89
Item 58	0.85	0.78	0.87	0.82
Affective disturbance (18 items)				
Item 1	0.83	0.67	0.85	0.69
Item 3 (r)	0.50	0.41	0.51	0.43
Item 6	0.77	0.56	0.79	0.58
Item 8	0.74	0.63	0.76	0.66
Item 10	0.66	0.65	0.67	0.68
Item 12	0.64	0.50	0.65	0.52
Item 13	0.77	0.58	0.79	0.61
Item 18	0.48	0.37	0.49	0.39
Item 22	0.75	0.54	0.77	0.56
Item 28 (r)	0.47	0.42	0.49	0.44
Item 30	0.66	0.51	0.67	0.53
Item 38	0.80	0.60	0.81	0.63
Item 43	0.88	0.78	0.89	0.81
Item 44	0.90	0.80	0.92	0.83
Item 45	0.87	0.76	0.89	0.79
Item 46	0.91	0.83	0.92	0.86
Item 50	0.67	0.47	0.69	0.50
Item 54	0.90	0.84	0.91	0.88
Loss of cognitive control (15 items)				
Item 5	0.71	0.57	0.74	0.62
Item 9	−0.11	0.03	−0.11	0.03
Item 11	0.70	0.59	0.73	0.64
Item 14	0.73	0.63	0.76	0.68
Item 17	0.78	0.71	0.81	0.76
Item 24 (r)	−0.62	.‐57	−0.65	−0.61
Item 26	0.85	0.69	0.88	0.74
Item 33	0.83	0.75	0.86	0.80
Item 34 (r)	−0.15	−0.16	−0.16	−0.18
Item 37 (r)	0.00	0.04	0.00	0.04
Item 48	0.85	0.75	0.89	0.81
Item 51	0.80	0.69	0.83	0.74
Item 57	0.75	0.69	0.78	0.74
Item 59	0.82	0.74	0.85	0.80
Item 61	0.75	0.75	0.78	0.81
Hyperarousal (13 items)				
Item 7	0.76	0.52	0.78	0.55
Item 16	0.84	0.65	0.86	0.70
Item 20	0.61	0.59	0.63	0.63
Item 21	0.71	0.50	0.73	0.53
Item 29	0.59	0.44	0.61	0.47
Item 32	0.58	0.52	0.59	0.56
Item 36	0.77	0.65	0.78	0.69
Item 41	0.75	0.65	0.76	0.70
Item 42	0.59	0.57	0.61	0.60
Item 47	0.82	0.67	0.84	0.71
Item 49	0.77	0.53	0.79	0.57
Item 53	0.72	0.63	0.74	0.67
Item 60	0.87	0.68	0.89	0.73
Social withdrawal (5 items)				
Item 23	0.78	0.65	0.87	0.79
Item 31	0.73	0.49	0.81	0.60
Item 40	0.76	0.59	0.84	0.72
Item 52	0.71	0.62	0.78	0.76
Item 55	0.78	0.61	0.86	0.74

*Note:* All factor loadings were significant at level *p* < 0.001, except for Item 37r in the online sample. Three reverse‐coded Items 24r, 34r, 37r, and 9 were excluded for modified CFA models due to poor factor loadings and inconsistency with theory.

**TABLE 4:** Standardized covariances between all latent factors of the SCI‐2‐G.

Variable	1	2	3	4
Online sample				
1. Entrapment	—			
2. Affective disturbance	0.95	—		
3. Loss of cognitive control	0.90	0.90	—	
4. Hyperarousal	0.92	0.95	0.95	—
5. Social withdrawal	0.86	0.87	0.79	0.86
Outpatient sample				
1. Entrapment	—			
2. Affective disturbance	0.90	—		
3. Loss of cognitive control	0.80	0.82	—	
4. Hyperarousal	0.80	0.87	0.93	—
5. Social withdrawal	0.76	0.80	0.66	0.72

*Note:* All covariances were significant at a level of *p* < 0.001.

#### 3.1.2 Outpatient Sample

The one‐factor model demonstrated acceptable but not ideal fit, with CFI = 0.974, TLI = 0.973, RMSEA = 0.081, SRMR = 0.075, and a significant chi‐square test (*χ* ^2^[1769] = 8614.20, *p* < 0.001). The five‐factor model exhibited adequate‐to‐good fit (CFI = 0.982, TLI = 0.981, RMSEA = 0.067, SRMR = 0.067), with a significant chi‐square statistic, as expected given the large sample size (*χ* ^2^[1759] = 6442.71, *p* < 0.001). All items loaded above 0.30 on their respective factors, except for the reverse‐coded Items 24r, 34r, 37r, and 9. Removing these items did not noticeably improve global model fit. The CFA supported both the one‐ and five‐factor solutions, with the five‐factor model demonstrating superiority in the outpatient sample (Δ*χ* ^2^[10] = 2171.49, *p* < 0.001). Standardized factor loadings and significant covariances are shown in Tables 3 and 4.

Overall, the CFA results provide strong support for the five‐factor solution over the one‐factor solution. However, reverse‐coded items (24r, 34r, and 37r) and Item 9 showed poor loadings across all analyses and their removal did not improve global model fit.

### 3.2 Internal Consistency

The internal consistency of the German revised version SCI‐2‐G was assessed by calculating Cronbach's *α* for both its total score and subscale scores of each dimension. Both in the online and outpatient sample, the total score demonstrated excellent internal consistency (online: Cronbach's *α* = 0.98; outpatient: *α* = 0.96). Three subscales showed good to excellent internal consistency in both samples: *entrapment* (online: *α* = 0.95; outpatient: *α* = 0.92), *affective disturbance* (online: *α* = 0.94; outpatient: *α* = 0.90), and *hyperarousal* (online: *α* = 0.92; outpatient: *α* = 0.85). In contrast, two subscales demonstrated acceptable to good reliability: *loss of cognitive control* (online: *α* = 0.85; outpatient: *α* = 0.79) and *social withdrawal* (online: *α* = 0.88; outpatient: *α* = 0.79).

### 3.3 Convergent and Current Criterion Validity

Convergent validity was supported by strong, significant correlations between the German SCI‐2 and severity of depression, assessed with the PHQ‐9 in the online sample and the BDI‐II and DASS‐D in the outpatient sample (see Table 5). Anxiety (DASS‐A) and stress (DASS‐S) also strongly correlated with the SCI‐2 in the outpatient sample. Concurrent criterion validity was supported by moderate significant correlations between the SCI‐2‐G and past‐month suicidal ideation. Point‐biserial correlations with lifetime suicide attempts were significant but weak to moderate.

**TABLE 5:** Correlations between SCI‐2‐G total/subscale scores and other relevant instruments for convergent and concurrent criterion validity.

	SCI‐2‐G total score	SCI‐2‐G entrapment	SCI‐2‐G affective disturbance	SCI‐2‐G Loss of cognitive control	SCI‐2‐G hyperarousal	SCI‐2‐G social withdrawal
PHQ‐9 (online)	0.85***	0.80***	0.81***	0.75***	0.81***	0.72***
BDI‐II (outpatients)	0.72***	0.66***	0.66***	0.59***	0.62***	0.61***
DASS‐D (outpatients)	0.70***	0.72***	0.68***	0.53***	0.53***	0.61***
DASS‐A (outpatients)	0.63***	0.49***	0.64***	0.55***	0.58***	0.41***
DASS‐S (outpatients)	0.69***	0.54***	0.59***	0.63***	0.74***	0.48***
SI (online)	0.69***	0.69***	0.67***	0.58***	0.62***	0.59***
SI (outpatients)	0.43***	0.47***	0.42***	0.28***	0.32***	0.36***
SA life (online)	0.40***	0.40***	0.41***	0.32***	0.38***	0.33***
SA life (outpatients)	0.20**	0.18***	0.20***	0.13**	0.17***	0.20***

*Note:* Correlations were significant at level **p < 0.01 or ***p < 0.001. In the online sample, depression was assessed with the PHQ‐9. In the outpatient sample, depression was assessed with the BDI‐II and DASS‐D, anxiety and stress with the DASS‐A and DASS‐S. In both samples, past‐month suicidal ideation (SI) and lifetime suicide attempts (SA life) were assessed with the SIBS.

## 4 Discussion

The purpose of this study was to evaluate the psychometric properties of the German revised SCI (SCI‐2‐G) in an online and outpatient sample. To our knowledge, this is the first evaluation of the scale's psychometric properties in Germany. In accordance with our first hypothesis and previous literature (Bloch‐Elkouby et al. 2021; Menon et al. 2022; Park et al. 2023), the CFA indicated adequate‐to‐good to good model fit for one‐ and five‐factor solutions in the online and outpatient samples. One possible explanation for the rather adequate fit may be the sample sizes, which did not exceed Nunnally's (1978) recommended item‐to‐respondent ratio of at least 10 participants per item (i.e., 610 for 61 items) for CFA.

Overall, the five‐factor solution consistently outperformed the one‐factor solution across both samples, providing support for the factorial validity of the SCI‐2‐G in Germany. Notably, this finding differs from the earlier German study of forensic inpatients which could not replicate the five‐factor structure using an older version of the instrument (SCI‐G; Otte et al. 2020). Our results suggest that the German version of the revised SCI (SCI‐2‐G) is suitable for measuring this suicide‐specific syndrome in the German population. The findings support both its five‐factor structure—despite limited psychometric performance of the *loss of cognitive control* dimension—and its unidimensional character as proposed by the authors. Our results contribute to the cross‐cultural perspective of the SCS. However, no conclusions can be drawn about its predictive validity or its actual ability to indicate a suicidal crisis.

The second hypothesis with regard to reliability was supported by an excellent internal consistency of the total scale in both samples (online: *α* = 0.98; outpatient: *α* = 0.96) and of the subscales *entrapment*, *affective disturbance*, and *hyperarousal*, consistent with the literature in diverse samples (e.g., Bloch‐Elkouby et al. 2021; Menon et al. 2022; Wu et al. 2022). The subscales *loss of cognitive control* (online: *α* = 0.85; outpatient: *α* = 0.79) and *social withdrawal* (online: *α* = 0.88; outpatient: *α* = 0.79) demonstrated acceptable to good reliability, also reported by other authors (Chistopolskaya et al. 2022; Wu et al. 2022; Peper‐Nascimento et al. 2024).

Moreover, the reverse‐coded Items 24r, 34r, and 37r, as well as Item 9, consistently demonstrated poor factor loadings onto the cognitive rigidity scale of the *loss of cognitive control* factor. Similarly, poor factor loadings for these reverse‐coded items have been observed in other CFA studies, suggesting either weak construct validity of this subscale or imprecise item formulations (Menon et al. 2022; Park et al. 2023; Peper‐Nascimento et al. 2024). Peper‐Nascimento et al. (2024) reported improved model fit after removing these items, highlighting potential cultural differences in the role of rigidity during a suicidal crisis and recommending a cross‐cultural adaptation of the subscale. In contrast, we found no improvement in model fit after their removal. Given the poor factor loadings and additional findings, we suggest reconsidering the inclusion of these items in the instrument.

Rather than indicating an acute suicidal crisis, aspects of this dimension—such as cognitive rigidity and (suicide‐specific) rumination—may also reflect a long‐term risk for suicidal thoughts and behavior (Rogers and Joiner 2018b), as suggested by the metacognitive model of suicidality (see Forkmann et al. 2025). The reverse‐coded SCI‐2 items (e.g., Item 37r—“Did you feel you could easily change your mind over things that bother you?”) demonstrated high semantic similarity to items in scales that measure cognitive rigidity and rumination such as the Suicide Rumination Scale (SRS; Rogers et al. 2022; e.g., “When I have thoughts of suicide, I am unable to stop thinking about suicide”) or the Perseverative Thinking about Suicide Questionnaire (PTSQ; Höller et al. 2022; e.g., “My thoughts about suicide repeat themselves”). For example, evidence shows that suicide‐specific rumination is associated with suicidal intent across time in the United States (Rogers, Gallyer, and Joiner 2021) and predictive of suicide planning and intent in Germany (Hensel et al. 2024). Future studies should explore whether cultural differences in item interpretation—especially between Eastern and Western cultures (De Vaus et al. 2018; cited by Park et al. 2023)—or factors like the lower base rate of suicidality (and potentially less cognitive rigidity) in community samples contribute to the weaker reliability observed in Brazil (Peper‐Nascimento et al. 2024) and this study as compared to findings from the United States (Bloch‐Elkouby et al. 2021). The weaker correlations of the cognitive rigidity items with the rest of the SCI‐2 may also stem from limitations in item formulation that do not adequately reflect the construct. Future studies should carefully examine whether this reflects an issue of operationalization or a more fundamental problem with the construct itself.

With regard to the third hypothesis, convergent validity was supported by moderate to strong positive correlations between the SCS total/subscales and related constructs such as depression, anxiety, and stress. Concurrent criterion validity was also demonstrated by moderate but significant correlations between the SCI‐2‐G and past‐month suicidal ideation, in line with findings ranging from *r* = 0.33, *p* < 0.01 in Korea (Park et al. 2023) to *r =* 0.54, *p* < 0.001 in Brazil (Peper‐Nascimento et al. 2024). It is somewhat surprising that correlations with lifetime suicide attempts were weak to moderate, in contrast to the larger moderate correlations for the original SCI (Galynker et al. 2017). Yet, weak correlations with the SCI‐2 have also been reported by other authors (e.g., *r* = 0.15, *p* = 0.040, Bloch‐Elkouby et al. 2021). A possible explanation for the weaker correlation with lifetime suicide attempts may be the time frame of the SCS construct, which assesses acute suicide risk and thus demonstrates stronger concurrent and near‐term predictive validity. Consequently, it may not align with retrospective measures of past suicidal behavior. Although a higher correlation with lifetime attempts could suggest that the SCS reflects a recurrent acute state, such recurrence may not be predictable, especially in a sample from online and routine outpatient settings, where the base rate of prior suicide attempts is relatively low.

### 4.1 Limitations

On the one hand, a strength of this study was the inclusion of two distinct samples from the population: one comprising individuals receiving routine outpatient psychotherapeutic care and another consisting of a rather nontreatment‐seeking sample. Interestingly, these sociodemographic characteristics linked to more severe SCS scores (Rogers, McMullen, et al. 2023) might explain the higher presence of SCS above the cutoff in the overall sample (16.8%) compared to Rogers's cross‐national findings. This broadens the scope of previous analyses and sets the studied group apart from primarily community‐focused validation studies (e.g., Menon et al. 2022; Wu et al. 2022; Peper‐Nascimento et al. 2024). Notably, the suggested cutoff score was established using a hospital‐based clinical sample comprising both inpatients and outpatients (Bloch‐Elkouby et al. 2021) and has not yet been validated in other populations. The relatively high prevalence observed in our sample (> 15%) may indicate limitations in its generalizability.

On the other hand, not all questionnaires were assessed across both samples, limiting the overall analysis and making certain pooled correlation analyses impossible. In addition, recruitment strategies differed in that outpatients were recruited as they began therapy, whereas the online sample was recruited via a flyer titled “Online Survey on Protective and Risk Factors for Suicidal Experiences.” This wording may have resonated more strongly with individuals who had previously experienced suicidal thoughts or behaviors. The findings should therefore be interpreted with caution, as this self‐selection may partly explain the higher prevalence of concurrent suicidal ideation and previous suicide attempts in the online sample compared to the outpatient sample.

Another limitation is the cross‐sectional design, with no longitudinal data available to examine the predictive validity of the SCI‐2‐G in this population. While prior studies reported good model fit, our individual results indicate adequate‐to‐good fit. This discrepancy could be attributed to differences in sample characteristics, measurement approaches, or fit indices used. If the same cutoff scores for indicating good model fit were used as in Bloch‐Elkouby et al.'s (2021) study, the interpretation of the results would be slightly different. These potential limitations highlight the importance of considering population and contextual variability when evaluating model fit across studies.

### 4.2 Clinical Implications

Although the confirmed five‐factor structure of the SCS supports the conceptualization of the proposed diagnosis, the clinical utility of a 61‐item questionnaire in suicidal patients is questionable. Thus, shorter instruments have already been developed, including a brief five‐symptom clinician‐rated diagnostic tool, the SCS Checklist (SCS‐C; Bafna et al. 2022), an abbreviated SCS Checklist (A‐SCS‐C; Karsen et al. 2023; Cohen et al. 2024) for risk assessment in the emergency department with two screening items, and an eight‐item short form for use in clinical settings (SCI‐2‐SF; De Luca et al. 2024), which has already been validated in Germany (Spangenberg et al. 2025). In addition, an abbreviated version of the five‐domain SCI‐2 is currently being developed by one of the co‐authors. Future studies on their predictive validity and clinical utility have yet to be conducted.

## 5 Conclusion

Findings initially support the five‐factor structure of the SCS in Germany, highlighting its potentially greater clinical and diagnostic utility compared to a one‐factor solution. Although previous studies reported strong model fit, the CFA in this study demonstrated adequate‐to‐good fit depending on the sample and indices used. Future research should further explore the cross‐cultural perspective through longitudinal studies in diverse populations to enhance generalizability and clinical application.

## Author Contributions

**Laura Melzer:** writing – original draft, conceptualization, data curation, methodology, formal analysis. **Carola Claus:** writing – review and editing, validation. **Nelia Posen:** data curation, investigation, methodology. **Thomas Forkmann:** writing – review and editing, supervision. **Megan L. Rogers:** methodology, writing – review and editing. **Tobias Teismann:** conceptualization, writing – review and editing, supervision.

## Ethics Statement

The Ethics Committee of the Faculty of Psychology at Ruhr University Bochum approved the data collection. All participants provided informed consent.

## Conflicts of Interest

The authors declare no conflicts of interest.

## Data Availability

The data that support the findings of this study are available from the corresponding author upon reasonable request.
